# Evaluation of rectum and bladder dose accumulation from external beam radiotherapy and brachytherapy for cervical cancer using two different deformable image registration techniques

**DOI:** 10.1093/jrr/rrx028

**Published:** 2017-06-08

**Authors:** Noriyuki Kadoya, YuYa Miyasaka, Takaya Yamamoto, Yoshihiro Kuroda, Kengo Ito, Mizuki Chiba, Yujiro Nakajima, Noriyoshi Takahashi, Masaki Kubozono, Rei Umezawa, Suguru Dobashi, Ken Takeda, Keiichi Jingu

**Affiliations:** 1 Department of Radiation Oncology, Tohoku University Graduate School of Medicine, 1–1 Seiryo-machi, Aoba-ku, Sendai, 980–8574, Japan; 2 Department of Mechanical Science and Bioengineering, Graduate School of Engineering Science, Osaka University, Toyonaka, Japan; 3 Department of Radiological Technology, School of Health Sciences, Faculty of Medicine, Tohoku University, Sendai, Japan

**Keywords:** radiotherapy, brachytherapy, deformable image registration, dose accumulation, cervical cancer

## Abstract

We evaluated dose–volume histogram (DVH) parameters based on deformable image registration (DIR) between brachytherapy (BT) and external beam radiotherapy (EBRT) that included a center-shielded (CS) plan. Eleven cervical cancer patients were treated with BT, and their pelvic and CS EBRT were studied. Planning CT images for EBRT and BT (except for the first BT, used as the reference image) were deformed with DIR to reference image. We used two DIR parameter settings: intensity-based and hybrid. Mean Dice similarity coefficients (DSCs) comparing EBRT with the reference for the uterus, rectum and bladder were 0.81, 0.77 and 0.83, respectively, for hybrid DIR and 0.47, 0.37 and 0.42, respectively, for intensity-based DIR (*P* < 0.05). D_1 cm^3^_ for hybrid DIR, intensity-based DIR and DVH addition were 75.1, 81.2 and 78.2 Gy, respectively, for the rectum, whereas they were 93.5, 92.3 and 94.3 Gy, respectively, for the bladder. D_2 cm^3^_ for hybrid DIR, intensity-based DIR and DVH addition were 70.1, 74.0 and 71.4 Gy, respectively, for the rectum, whereas they were 85.4, 82.8 and 85.4 Gy, respectively, for the bladder. Overall, hybrid DIR obtained higher DSCs than intensity-based DIR, and there were moderate differences in DVH parameters between the two DIR methods, although the results varied among patients. DIR is only experimental, and extra care should be taken when comparing DIR-based dose values with dose–effect curves established using DVH addition. Also, a true evaluation of DIR-based dose accumulation would require ground truth data (e.g. measurement with physical phantom).

## INTRODUCTION

Brachytherapy (BT) has played an essential role in the treatment of gynecologic malignancies for decades. Locally advanced cervical cancer is treated with a combination of concomitant chemotherapy, external beam radiotherapy (EBRT) and a BT boost to the cervical regions [1–5]. Recently, 3D image-guided brachytherapy (3D-IGBT) has been widely employed for cervical cancer, resulting in dose–volume histogram (DVH)-based evaluation [1, 3, 6, 7]. Recommendations for 3D-IGBT for cervical cancer were published by the working group for gynecologic brachytherapy of the Groupe Européen de Curiethérapie–European Society for Radiotherapy and Oncology (GEC-ESTRO) and have become standard practice in many institutions [8–11].

GEC–ESTRO recommended adding DVH parameters from each BT fraction and EBRT in equivalent doses in 2 Gy fractions (EQD2). In general, all the DVH parameters were added using DVH parameter addition, based on the assumption that the hotspots for the target were located in the same regions of the target and organs at risk (OARs) in each treatment. To improve the accuracy of DVH-based evaluation, several recent studies have investigated a dose accumulation method for evaluating the DVH parameters based on deformable image registration (DIR) [12–14]. Andersen *et al.* reported a >5% dose difference for bladder D_0.1 cm^3^_ between simple DVH parameter addition and DIR-based dose accumulation in 38% of the patients studied [12]. Abe *et al.* also showed that DIR between the planning CT scan for the first BT and that of other BT fractions could achieve reasonable DIR accuracy with Dice similarity coefficients (DSCs) of ~0.8 for the high-risk clinical target volume (HR-CTV), rectum and bladder, suggesting that DIR-based dose accumulation may be acceptable for assessing accumulated dose distributions in combined radiotherapy for cervical cancer [13]. These previous studies focused on the DVH-based dose accumulation between each BT fraction, and to date there have been few published reports that evaluate DIR between EBRT and BT using DIR algorithms [15]. Teo *et al.* evaluated the accumulated dose of BT and EBRT with or without boost plan in 20 cervical cancer patients. They suggested that DVH parameter addition can be used as a good approximation of D_2 cm^3^_ when adding BT and EBRT doses. However, they did not sufficiently evaluate the accumulated dose for BT and EBRT.

In terms of DIR accuracy, DIR between the planning CTs for EBRT and for BT is challenging due to the considerable deformation caused by the intracavity applicator in BT. Commercial DIR software is available for use in clinical practice, including MIM Maestro (MIM Software Inc., Cleveland, USA), Velocity (Varian Medical Systems, Palo Alto, USA) and RayStation (RaySeach Laboratories, Stockholm, Sweden) [16–18]. In these software packages, an intensity-based DIR algorithm (i.e. DIR without structure information) is commonly used. It is expected that this DIR would not result in good registration accuracy when the deformation is large or when the boundaries between structures are not clear. To improve DIR accuracy, hybrid DIR has been recently developed and implemented in RayStation [19]. This has the potential to improve DIR accuracy, even with large deformation [20].

Thus, in this study, first, we evaluated the DIR accuracy between the planning CT images for EBRT and BT using DSC for intensity-based and hybrid DIR methods. Then, we clarified the difference in DIR-based dose accumulation between the two DIR methods.

## MATERIALS AND METHODS

### Patient characteristics

This study was a retrospective analysis approved by our institutional review board (2015-1-167). Eleven consecutive patients with cervical cancer treated at our hospital in the period from January 2015 to June 2015 were selected for this study. In six of the patients, the cancer was classified as International Federation of Gynecology and Obstetrics (FIGO) Stage IIB, in three patients it was classified as Stage IB, and in the other two patients it was classified as Stage IIIB. All the patients received EBRT to the whole pelvis (median 30 Gy with range 20–30 Gy). In addition, all patients underwent CS EBRT with 4 cm width at the midline to avoid overdose to the rectum and bladder (median, 20 Gy; range, 10–30 Gy). For all patients, the total dose of EBRT was 50 Gy. The EBRT plan was created with the 4-field box technique with 10 MV X-rays, and the CS EBRT plan was created with the anterior–posterior/posterior–anterior (AP/PA) parallel-opposed field technique with a midline block to avoid overdose in the zone where the rectum and bladder receive the highest BT dose. This technique is commonly used in Japan [21]. The dose calculation algorithm was the Anisotropic Analytical algorithm implemented in Eclipse version 11.0 (Varian Medical Systems, Palo Alto, USA). All the patients received ^192^Ir high-dose-rate BT once per week for four consecutive weeks, concurrently with the CS EBRT. A Fletcher CT-MR applicator (Elekta, Stockholm, Sweden) was used in each case. After the applicators were inserted into the vagina, gauze was packed on the anterior and posterior sides of the applicators. The minimum dose delivered to 90% of the most irradiated volume of the high-risk CTV (HR-CTV D90) and the minimum dose delivered to D_2 cm^3^_ of the rectum and the bladder were calculated. At least 6 Gy was prescribed to the HR-CTV D90 in each BT session. The dose constraint was 75 Gy in D_2 cm^3^_ of the rectum and 90 Gy in D_2 cm^3^_ of the bladder. The CS plan was not considered for DVH parameter addition because the CS plan fields completely blocked the radiation to the regions where the rectum and bladder receive the highest BT dose. For example, when the EBRT dose is 50 Gy (WP:30 Gy, CS:20 Gy), we assume that the EBRT dose is 30 Gy (without considering the CS dose).

The treatment planning system used for BT (to design the CT-based treatment plan) was Oncentra version 4.1 (Elekta, Stockholm, Sweden).

### DIR-based dose accumulation

**Fig. 1.**
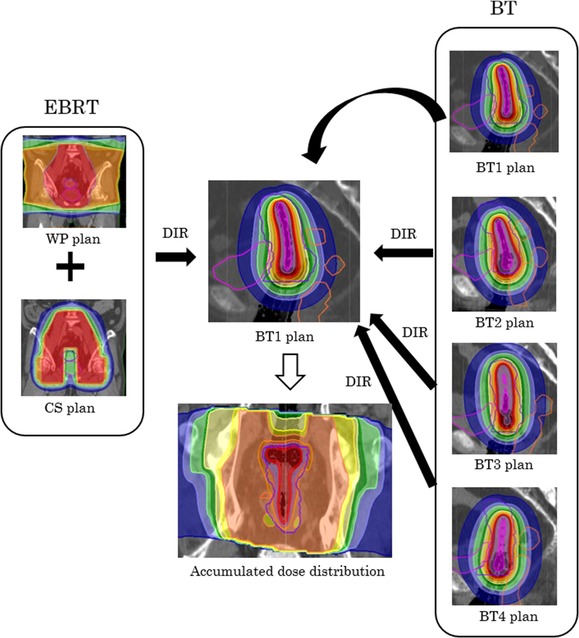
A schematic diagram of the creation of the accumulated dose distribution using deformable image registration (DIR). Planning CT images for external beam radiotherapy (EBRT) and brachytherapy (BT) (except for the first BT) were deformed with DIR to the CT image of the first BT (reference image). EBRT consisted of the whole pelvis plan (WP plan) and the center-shielded boost plan (CS plan). We then calculated the accumulated dose between the plans on the reference image.

A process workflow for creating the DIR-based dose accumulation is shown in Fig. 1. The process used the planning CT image for the first day of BT as the reference image.

First, both planning CT image sets (with and without shielding) were rigidly fused by matching the uterus structures to the reference image by manually shifting and rotating the images. Next, the planning CT images for BT of the four BT fractions (except for the first day’s image) were rigidly fused by matching the uterus structures to the reference image by manually shifting and rotating the images, referring to the applicator position in the reference image. Then, the planning CT images for EBRT and BT (except for the first day) were deformed to the reference image (first BT fraction) using DIR. We used hybrid intensity and structure-based deformable image registration (the ANACONDA algorithm) implemented in RayStation version 4.5.1 (RaySearch Laboratories, Stockholm, Sweden). This algorithm combines image information (i.e. intensities) with anatomical information as provided by contoured image sets [19]. To evaluate the effect of an approach that combined the intensity-based and anatomical information-based approaches for the improvement of DIR accuracy, two different DIR parameter settings were employed. One setting used the only whole-body structure for DIR (i.e. the whole body structure was used to define the registration region). This setting assumes that this registration uses the intensity-based approach (intensity-based DIR). The other setting used the uterus, rectum, bladder and body structures for DIR, assuming that this registration is based on both the intensity-based and anatomical information-based approaches (hybrid DIR). Before DIR was performed, we performed image processing to improve the DIR accuracy: the CT values within applicators were replaced by −1000 HU by in-house software programmed by MATLAB 2011a (Mathworks, MA, USA). After DIR, the deformation fields derived from the DIR were applied to the dose distributions for EBRT and BT for accumulation purposes.

### Evaluation of the accumulated DVH parameters from the two calculation methods: DIR-based and DVH parameter addition-based

The DIR-based dose accumulation was calculated as described above, and the minimum doses to the most exposed 0.1-, 1- and 2-cm^3^ tissue (D_0.1 cm^3^_, D_1 cm^3^_ and D_2 cm^3^_) for the rectum and bladder were calculated using accumulated dose distribution. The DVH parameters were calculated by DVH parameter addition, adding the components of the EBRT and BT sessions. This method is recommended by GEC–ESTRO and is based on the assumption that the locations of the most exposed volumes were identical at each BT fraction. It should be noted that we calculated the accumulated DVH for EBRT and BT for the rectum and bladder after dose distributions, with the physical dose for both treatments converted to the equivalent dose in 2 Gy fractions (EQD2) according to the linear quadratic model using α/β = 3 Gy [22]. For the EBRT dose, the CS plan was not considered for DVH parameter addition because the CS plan fields completely blocked the radiation to the regions where the rectum and bladder receive the highest BT dose, as mentioned in the previous section.

### Quantitative evaluation of image registration accuracy

The DSC was calculated to evaluate registration accuracy. The DSC is designed to evaluate quantitatively the two sets of contours [23, 24] and is defined as

(1) $$\mathrm{DSC}=\frac{V_{d}\cap V_{s}}{(V_{d}+V_{s})/2},$$

where *V*_d_ is the volume of the structure deformed by DIR (or of a structure transformed by rigid registration) and *V*_s_ is the volume of the contours manually delineated on the planning CT image of the reference image. A DSC value of 0 indicates no spatial overlap, and a value of 1 indicates perfect agreement between the two contoured volumes.

### Statistical analysis

Tukey’s honestly significant difference test was used to compare the mean DSCs and the mean D_2 cm^3^_, D_1 cm^3^_ and D_0.1 cm^3^_ for the rectum and bladder. All tests were two-sided, with *P* < 0.05 considered significant. Statistical analysis was performed with JMP version 11.2 (SAS Institute, Cary, NC).

## RESULTS

### Evaluation of registration accuracy

**Fig. 2.**
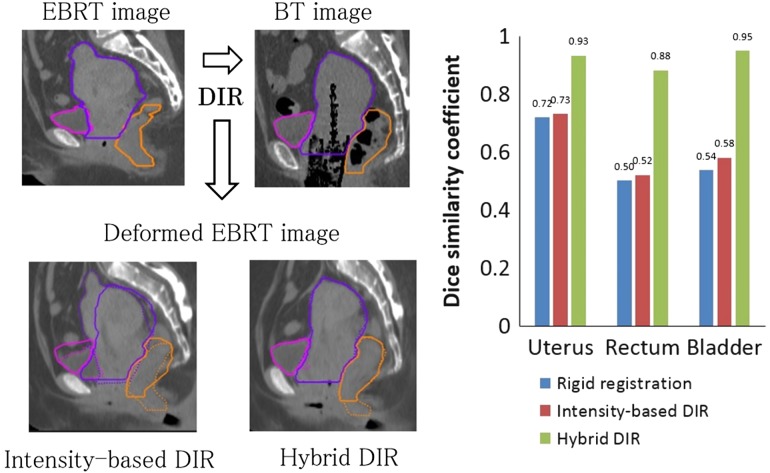
Comparison of the EBRT CT image deformed by intensity-based deformable image registration (DIR) with that by hybrid DIR for Patient 3, showing that the largest Dice similarity coefficient (DSC) was between EBRT and BT for the uterus. In the deformed EBRT CT image, purple indicates the uterus, orange the rectum, and pink the bladder. A solid line represents manually delineated structures and a dotted line represents structures created by DIR.

The mean DSCs for rigid registration, intensity-based DIR and hybrid DIR between the EBRT or each BT fraction and the reference image are shown in Table 1. These results showed that for all structures, hybrid DIR had the best registration accuracy (intensity vs hybrid, *P* < 0.05 for all structures) and an approximate DSC value of 0.8. Registration accuracy between BT images was higher than that between EBRT and BT for all structures (e.g. uterus with hybrid DIR, 0.81 vs 0.93), suggesting that the relatively large deformation caused by the applicator reduced the registration accuracy. Figure 2 shows typical examples of the deformed images created by the two DIR methods for Patient 3, showing the largest DSC value was for hybrid DIR in the uterus. From visual inspection, the manually delineated contour and the contour created by hybrid DIR had good agreement for all structures (DSCs: uterus, 0.93; rectum, 0.88; bladder, 0.95), compared with the results for intensity-based DIR (DSCs: uterus, 0.73; rectum, 0.52; bladder, 0.58), indicating that anatomical information was useful for improving DIR accuracy.

**Table 1.** Dice similarity coefficients (mean ± SD) for rigid registration, intensity-based DIR, and hybrid DIR between the EBRT for each BT fraction and the reference image (the first BT)

		Uterus	Rectum	Bladder
EBRT→BT1	Rigid registration	0.57 ± 0.11	0.33 ± 0.13	0.37 ± 0.18
	Intensity-based DIR	0.47 ± 0.19	0.37 ± 0.16	0.42 ± 0.19
	Hybrid DIR	0.81 ± 0.11	0.77 ± 0.09	0.85 ± 0.15
	*P* value (Rigid vs Intensity)	0.24	0.77	0.83
	*P* value (Rigid vs Hybrid)	0.002	<0.001	<0.001
	*P* value (Intensity vs Hybrid)	<0.001	<0.001	<0.001
Average (BT only)	Rigid registration	0.74 ± 0.05	0.53 ± 0.13	0.44 ± 0.17
	Intensity-based DIR	0.75 ± 0.05	0.57 ± 0.11	0.53 ± 0.19
	Hybrid DIR	0.93 ± 0.05	0.81 ± 0.16	0.80 ± 0.23
	*P* value (Rigid vs Intensity)	0.84	0.81	0.55
	*P* value (Rigid vs Hybrid)	<0.001	<0.001	<0.001
	*P* value (Intensity vs Hybrid)	<0.001	0.002	0.01

EBRT = external beam radiotherapy, BT = brachytherapy, DIR = deformable image registration, BT1 = first BT.

### Evaluation of DVH parameters

**Fig. 3.**
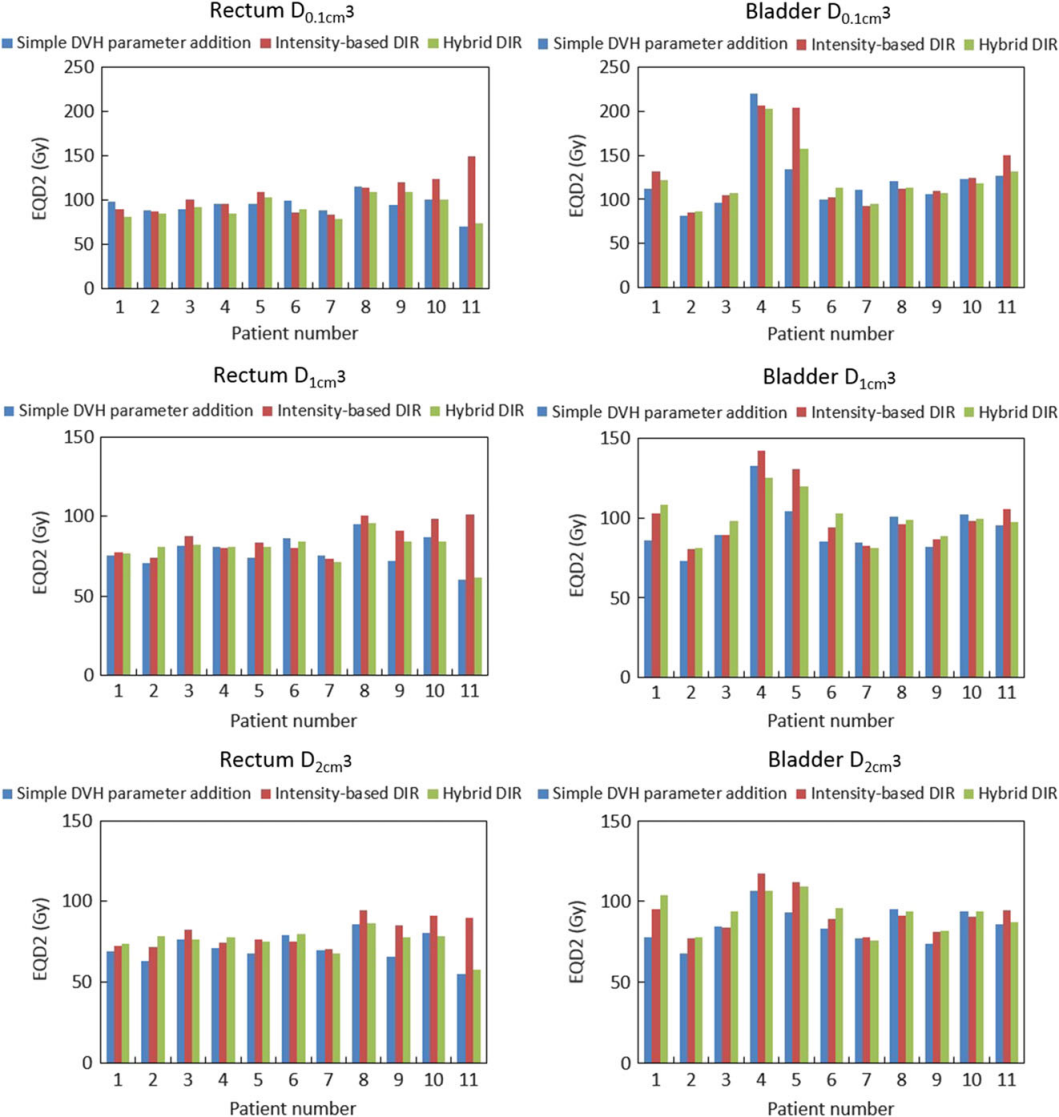
Mean D_0.1 cm^3^_, D_1 cm^3^_ and D_2 cm^3^_ of the rectum and bladder for BT and EBRT with a CS plan using DVH parameter addition, intensity-based DIR and hybrid DIR for individual patients.

First, the mean values of D_0.1 cm^3^_, D_1 cm^3^_ and D_2 cm^3^_ for the rectum and bladder with DVH parameter addition, intensity-based DIR, and hybrid DIR in combined BT and EBRT are shown in Fig. 3. Although the results varied among the patients, higher values of D_0.1 cm^3^_, D_1 cm^3^_ and D_2 cm^3^_ for the rectum and bladder with DVH parameter addition were seen than with DIR-based dose accumulation for almost all patients. The difference between DIR-based dose accumulation and DVH parameter addition was small for D_2 cm^3^_, but was large for D_0.1 cm^3^_. This was probably because DVH parameter addition resulted in an overestimation due to the lack of overlap between the CS and other plans.

**Fig. 4.**
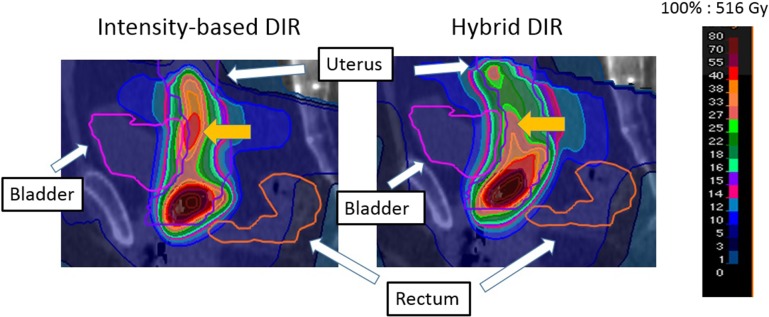
Comparison of the accumulated dose distributions with intensity-based DIR and with hybrid DIR, showing the large difference in bladder D_1 cm^3^_ between the two DIR methods. The large dose difference within the bladder is indicated by the yellow arrows.

Comparing intensity-based DIR and hybrid DIR, there was moderate difference between intensity-based and hybrid DIR in almost all patients. Individual differences between them exceeding 10 Gy were observed in several patients. The direction of this difference varied between patients, i.e. the DVH parameter values were higher with intensity-based DIR than with hybrid DIR for some patients, and vice versa for the others; overall, these differences may have canceled out. Figure 4 shows an example dose distribution for Patient 4, showing the large dose difference between intensity-based and hybrid DIR in D_1 cm^3^_ for the bladder (intensity-based DIR, 142.3 Gy vs hybrid DIR, 125.6 Gy). DSCs for the bladder for intensity-based and hybrid DIR were 0.25 and 0.57, respectively. This difference in DSC between the two DIR methods caused the large dose difference in D_1 cm^3^_ of bladder. These results suggested that DIR accuracy affected the accuracy of the accumulated dose.

It should be noted that the residual DIR error might cause a higher accumulated dose than simple DVH parameter addition, resulting in higher D_0.1 cm^3^_, D_1 cm^3^_ and D_2 cm^3^_ for DIR-based dose accumulation. For example, due to the residual DIR error, DIR-based dose accumulation with intensity-based DIR resulted in 84.2 Gy of rectum D_2 cm^3^_, compared with 55.2 Gy for DVH addition (Patient 11).

Next, the mean values of D_0.1 cm^3^_, D_1 cm^3^_ and D_2 cm^3^_ for the rectum and bladder with DVH parameter addition, intensity-based DIR and hybrid DIR are shown in Table 2. Although there was no significant difference between intensity-based and hybrid DIR, large differences were seen in some DVH parameters, as shown in Fig. 4. This is due to different DIR accuracy between the two DIR algorithms. The differences among dose accumulation with intensity-based DIR, dose accumulation with hybrid DIR, and DVH addition were calculated (Table 3). For the analysis with BT alone, DIR-based dose accumulation was likely to result in lower D_0.1 cm^3^_, D_1 cm^3^_ and D_2 cm^3^_ values than DVH parameter addition for hybrid DIR. This is because the most irradiated region of each OAR varied between each fraction because of differences in applicator positioning and organ filling. On the other hand, the results with intensity-based DIR were higher for several DVH parameters (e.g. for rectum D_0.1 cm^3^_). The reason for this might be due to a larger DIR error with intensity-based DIR than with hybrid DIR. For the analysis with BT and EBRT, DIR-based dose accumulation produced higher D_0.1 cm^3^_, D_1 cm^3^_ and D_2 cm^3^_ values than DVH parameter addition (except for rectum D_0.1 cm^3^_ with hybrid DIR) (*P* < 0.05). In addition, Table 3 also shows the difference in DVH parameters between intensity-based and hybrid DIR. The smaller the evaluated region, the larger the difference between the two DIR methods. The reason for this might be that the residual DIR error, which did not affect the minimum accumulated dose in the large evaluation regions (i.e. D_2 cm^3^_), changed the minimum accumulated dose in the small evaluation regions (i.e. D_0.1 cm^3^_).

**Table 2.** Mean values for D_0.1 cm^3^_, D_1 cm^3^_ and D_2 cm^3^_ for the rectum and bladder with DVH parameter addition, intensity-based DIR, and hybrid DIR

	DVH parameter	DVH parameter addition (Gy)		Intensity-based DIR (Gy)		Hybrid DIR (Gy)	
		mean ± SD	95% confidence interval	mean ± SD	95% confidence interval	mean ± SD	95% confidence interval
BT only	Rectum D_0.1 cm^3^_	65.9 ± 10.8	(59.6–75.0)	72.9 ± 17.8	(58.0–88.0)	58.5 ± 10.4	(50.1–96.1)
	Rectum D_1 cm^3^_	48.5 ± 8.5	(43.8–60.0)	53.1 ± 10.0	(44.6–69.1)	46.6 ± 7.4	(44.2–52.2)
	Rectum D_2 cm^3^_	41.9 ± 7.4	(37.7–50.1)	46.1 ± 8.5	(37.8–58.8)	41.7 ± 6.4	(40.1–47.4)
	Bladder D_0.1 cm^3^_	92.6 ± 35.6	(69.7–104.5)	93.6 ± 42.6	(61.5–168.8)	88.5 ± 34.6	(62.4–124.3)
	Bladder D_1 cm^3^_	67.6 ± 15.8	(54.8–74.4)	64.8 ± 21.4	(52.4–95.3)	65.4 ± 16.4	(53.7–90.8)
	Bladder D_2 cm^3^_	57.9 ± 10.7	(47.7–65.1)	55.0 ± 15.0	(46.5–76.9)	57.7 ± 12.9	(49.9–80.9)
BT + EBRT	Rectum D_0.1 cm^3^_	94.6 ± 10.4	(89.2–101.4)	102.4 ± 18.0	(87.7–118.8)	87.4 ± 11.4	(77.5–102.2)
	Rectum D_1 cm^3^_	78.2 ± 9.1	(72.3–86.8)	81.2 ± 10.9	(72.0–97.3)	75.1 ± 9.0	(68.5–82.5)
	Rectum D_2 cm^3^_	71.4 ± 8.4	(66.2–80.5)	74.0 ± 10.1	(67.4–86.7)	70.1 ± 8.1	(62.7–76.8)
	Bladder D_0.1 cm^3^_	121.2 ± 34.3	(99.7–134.7)	123.2 ± 41.7	(90.9–200.1)	118.1 ± 32.3	(94.0–156.2)
	Bladder D_1 cm^3^_	94.3 ± 15.3	(84.7–104.3)	92.3 ± 19.8	(73.8–121.4)	93.5 ± 15.0	(84.1–118.6)
	Bladder D_2 cm^3^_	85.4 ± 10.7	(76.9–95.4)	82.8 ± 13.7	(68.2–102.5)	85.4 ± 12.0	(75.6–105.1)

DVH = dose–volume histogram, DIR = deformable image registration, CS plan = center-shielded plan, D_0.1 cm^3^_ = minimum doses to the most exposed 0.1 cm^3^ of tissue, D_1 cm^3^_ = minimum doses to the most exposed 1 cm^3^ of tissue, D_2 cm^3^_ = minimum doses to the most exposed 2 cm^3^ of tissue, EBRT = external beam radiotherapy, BT = brachytherapy.

**Table 3.** Mean difference in D_0.1 cm^3^_, D_1 cm^3^_ and D_2 cm^3^_ for the rectum and bladder among DVH parameter addition and two DIR-based dose accumulations for EBRT and all BT sessions

	DVH parameter	Difference (Gy)		
		DVH parameter addition—intensity-based DIR	DVH parameter addition—hybrid DIR	Hybrid DIR—intensity-based DIR
BT only	Rectum D_0.1 cm^3^_	−7.0 ± 23.7	7.4 ± 7.0	−14.4 ± 20.5
	Rectum D_1 cm^3^_	−4.6 ± 12.3	1.9 ± 5.2	−6.5 ± 12.0
	Rectum D_2 cm^3^_	−4.3 ± 9.0	0.1 ± 4.7	−4.4 ± 10.0
	Bladder D_0.1 cm^3^_	−1.0 ± 24.1	4.1 ± 11.1	−5.1 ± 14.9
	Bladder D_1 cm^3^_	2.8 ± 12.3	2.2 ± 9.8	0.6 ± 6.0
	Bladder D_2 cm^3^_	2.9 ± 9.3	0.2 ± 8.8	2.7 ± 4.0
BT + EBRT	Rectum D_0.1 cm^3^_	−11.0 ± 24.7	2.8 ± 9.0	−13.8 ± 20.5
	Rectum D_1 cm^3^_	−7.9 ± 12.3	−2.1 ± 5.1	−5.9 ± 11.8
	Rectum D_2 cm^3^_	−9.0 ± 9.9	−4.1 ± 5.4	−4.9 ± 10.1
	Bladder D_0.1 cm^3^_	−8.3 ± 22.8	−2.2 ± 11.9	−6.1 ± 14.7
	Bladder D_1 cm^3^_	−6.5 ± 9.1	−5.9 ± 9.3	−0.6 ± 7.7
	Bladder D_2 cm^3^_	−6.5 ± 7.4	−7.3 ± 8.2	0.9 ± 5.9

## DISCUSSION

DIR has become commercially available in the field of radiotherapy. DIR is an exciting and interesting technology for multimodality image fusion, anatomic image segmentation, dose accumulation, and lung functional imaging [16, 25–27]. Although dose accumulation between EBRT and BT is challenging due to the relatively large deformation resulting from the intracavity applicator and tumor regression, it is important to evaluate the dose accumulation of the rectum and bladder accurately.

In this study, we evaluated the accuracy of DIR between CT images of EBRT and each BT fraction using different DIR algorithms. Our results showed that hybrid DIR could perform DIR more accurately than intensity-based DIR (especially DIR between EBRT and BT). Abe *et al.* evaluated the accuracy of DIR implemented using the commercial software MIM Maestro for five patients and reported mean DSCs for the HR-CTV, rectum and bladder as 0.78, 0.76 and 0.87, respectively [13]. In comparison, mean DSCs between the BT images and the first BT image obtained in the present study for the uterus, rectum and bladder with hybrid DIR were 0.93, 0.81 and 0.80, respectively. In their study, pre-imaging preparations for the uterus, rectum and bladder were performed to minimize change in interaction to obtain better DIR accuracy. Although we did not strictly control the angle of the uterus, the bladder volume, or rectum gas in our hospital, DSCs in our study were comparable in value (≥0.8). Additionally, two recently published studies reported a higher DSC value for OAR (e.g. DSC of rectum: 0.91) [15, 28]. They replaced the CT value within the OAR by 0 HU. Although this method is useful for improving the DSC value, this method is only focused on the shape of the specific structure, so a large DIR error might occur within the structure. Thus, the hybrid DIR algorithm used in our study has the potential for obtaining better DIR accuracy within the structure due to use of image intensity and anatomical information. There has been no definite consensus about the clinically meaningful DSC value. Macchia *et al.* reported that DSCs in prostate cancer patients for ABAS (Elekta, Stockholm, Sweden), MIM and Velocity AI were 0.77, 0.75 and 0.75, respectively, for rectum and were 0.93, 0.88 and 0.72, respectively, for bladder. Kirby *et al.* also evaluated the DIR accuracy with a pelvis phantom using 11 DIR algorithms and showed that the mean DSC for the rectum was 0.83. Although the DIR-based dose accumulation is sensitive to DIR error in the anterior rectal wall and the posterior bladder wall, a DSC value of ~0.8 might be a reasonable level of DIR accuracy.

In terms of DIR-based dose accumulation for the BT-only section, Andersen *et al.* reported that the difference in bladder D_2 cm^3^_ between DVH parameter addition and DIR-based dose accumulation was 0.4 ± 0.3 Gy [12]. Our values for hybrid and intensity-based DIR were 0.2 ± 8.8 Gy and 2.9 ± 9.3 Gy, respectively. Although our result for hybrid DIR was similar to their result, our result for intensity-based DIR was slightly different. The reason for this was that intensity-based DIR may include a larger DIR error than hybrid DIR, resulting in a large difference in the DVH parameters between DIR-based dose accumulation and DVH parameter addition. Based on this result, since the DVH parameters are used to estimate the rectal and bladder toxicity in clinical practice, it is essential that we pay attention to the DIR accuracy for DIR-based dose accumulation. For example, the EQDs for DVH parameter addition and hybrid DIR were very similar, but that of intensity-based DIR was considerably higher than these in the case of the rectum D2_ cm^3^_ for Patient 11. In addition, DIR is only experimental, and extra care should be taken when comparing DIR-based dose values with dose–effect curves established using DVH parameter addition.

Consequently, we suggested that DVH parameter addition provides a good estimate for D_1 cm^3^_ and D_2 cm^3^_ in these situations. However, a large difference in D_0.1 cm^3^_ between DVH parameter addition and DIR-based dose accumulation was seen. The reason for this was due to a slight difference in the hotspot location between each fraction, although the impact of the slight difference on the analysis of D_1 cm^3^_ and D_2 cm^3^_ was negligible.

Next, with regard to DIR-based dose accumulation for BT and EBRT, DIR-based dose accumulations for hybrid DIR produced higher D_1 cm^3^_ and D_2 cm^3^_ values than DVH parameter addition (*P* < 0.05). The reason for this could be that our CS plan using a midline bock with 4 cm width of the multileaf collimator could not completely block the the regions irradiated by the BT, although this accumulated dose might include the uncertainty caused by residual DIR error. That is, there was a protruded portion of the rectum and bladder volume from the shielded regions. Comparing intensity-based DIR and hybrid DIR, there was a moderate difference between intensity-based and hybrid DIR in almost all patients, as mentioned in the Results section. This result clearly showed that DIR-based dose accumulation strongly depends on DIR accuracy. Especially for BT, even if the residual DIR error was small, a large accumulated dose error might occur, due to the sharp dose distribution of BT. Thus, we should pay attention to the DIR accuracy for DIR-based dose accumulation.

There are some limitations to this study. First, although hybrid DIR achieved higher DIR accuracy than intensity-based DIR, hybrid DIR had residual DIR error. The residual DIR error might cause inaccurate dose accumulation. Particularly, the addition of dose–volume parameters for BT is largely dependent on the dose to the anterior rectal wall and the posterior bladder wall. The quality of DIR in these regions is important. A validation study on the accumulated dose using a deformable gynecological phantom created by a 3D printer is ongoing. In addition, to obtain a more reliable DIR-based dose accumulation, further improvement of DIR accuracy is required. Second, we used only one CT image for EBRT; it is better to use CT images for each treatment fraction for an accurate accumulated dose.

## CONCLUSIONS

Overall, hybrid DIR obtained higher DSCs than intensity-based DIR, and there were moderate differences in D_0.1 cm^3^_, D_1 cm^3^_ and D_2 cm^3^_ of rectum and bladder between the two DIR methods, although the result varied among patients. Our result showed that DIR-based dose accumulation for BT strongly depends on DIR accuracy. DIR is only experimental, and extra care should be taken when comparing DIR-based dose values with the dose–effect curves established using DVH addition for BT. In addition, a true evaluation of DIR-based dose accumulation would require ground truth data (e.g. measurement with a physical phantom).
